# How Accurately Can Prostate Gland Imaging Measure the Prostate Gland Volume? Results of a Systematic Review

**DOI:** 10.1155/2019/6932572

**Published:** 2019-03-03

**Authors:** David R. H. Christie, Christopher F. Sharpley

**Affiliations:** ^1^GenesisCare, Inland Drive, Tugun, QLD 4224, Australia; ^2^Brain-Behaviour Research Group, University of New England, Armidale, NSW 2350, Australia

## Abstract

**Aim:**

The measurement of the volume of the prostate gland can have an influence on many clinical decisions. Various imaging methods have been used to measure it. Our aim was to conduct the first systematic review of their accuracy.

**Methods:**

The literature describing the accuracy of imaging methods for measuring the prostate gland volume was systematically reviewed. Articles were included if they compared volume measurements obtained by medical imaging with a reference volume measurement obtained after removal of the gland by radical prostatectomy. Correlation and concordance statistics were summarised.

**Results:**

28 articles describing 7768 patients were identified. The imaging methods were ultrasound, computed tomography, and magnetic resonance imaging (US, CT, and MRI). Wide variations were noted but most articles about US and CT provided correlation coefficients that lay between 0.70 and 0.90, while those describing MRI seemed slightly more accurate at 0.80-0.96. When concordance was reported, it was similar; over- and underestimation of the prostate were variably reported. Most studies showed evidence of at least moderate bias and the quality of the studies was highly variable.

**Discussion:**

The reported correlations were moderate to high in strength indicating that imaging is sufficiently accurate when quantitative measurements of prostate gland volume are required. MRI was slightly more accurate than the other methods.

## 1. Introduction

There are many clinical situations in in the management of prostate diseases in which the measurement of the prostate gland volume (PGV) has a role [1–3]. For some of these the measurement does not need a high level of accuracy and simply detecting that the prostate is enlarged can be sufficient. For example, if a general practitioner is considering the choice of medication when treating benign prostatic hyperplasia (BPH), more precise measurements of the PGV may be required in other situations, for example, to calculate prostate specific antigen (PSA) density. For radiation oncologists, the PGV is used to determine the suitability of prostate cancer patients for low dose rate brachytherapy and the number of brachytherapy seeds to order. In those situations, a more accurate measure of the PGV is required and is usually obtained by medical imaging methods.

A number of imaging methods have been used to estimate the PGV, including ultrasound (US), either transrectally or suprapubically (TRUS, SPUS), Computer Tomography (CT), and Magnetic Resonance Imaging (MRI). Although many publications have described their accuracy, these have never been systematically reviewed, making it difficult to compare them. Our aim was to review the literature in order to determine the accuracy of imaging as a measure of PGV in a future planned study of the effects of neoadjuvant androgen deprivation therapy (NADT).

## 2. Materials and Methods

The PRISMA, AMSTAR-2, and QUADAS-2 tools were adopted to ensure the quality of the review. However, in this case the imaging tests were not being used as diagnostic tests but as measuring tools, so not all of the criteria for these were relevant [4–6]. The proposal for the review was submitted for registration to PROSPERO [7], but the review was completed before a response was received. Ethics committee approval was not required and no funding was obtained for this study.

The patient populations studied were those men undergoing imaging of the prostate for any reason, including those attending health services for prostate conditions. The interventions to be reviewed were the US, CT, and MRI, recognising that variations existing in the way each of these can be used to measure PGV. All study designs were considered and the outcome was to be any quantitative measure of accuracy when compared against the reference standard, meaning* in vitro* measurement of the PGV after radical prostatectomy.

Multiple medical literature databases were accessed in August 2018, including CINAHL Plus, Embase, Medline, Pubmed, and ScienceDirect and were searched for abstracts containing the terms “prostate volume” and “imaging OR US OR CT OR MRI” and “prostatectomy”. No other review protocol or similar previous publication existed. Titles and abstracts were reviewed by both of the authors and relevant full text articles were obtained for further review. The results were then tabulated so that the range of results could be seen, including correlations, concordance, and tendencies to over- or underestimate. For each study the date of publication, the numbers of patients, and the average age of the patients were tabulated.

Although there were relevant articles published over a period of more than 50 years, we arbitrarily adopted a time limit of 22 years (since 1995), as we assumed that the extensive developments in the technology of the imaging and reference methods would render articles published before that time less relevant. Titles that were published only published in abstract form or relating to animal studies were also excluded. Several articles have compared the accuracy of the other less invasive imaging methods with the TRUS including SPUS, transperineal US, CT, and MRI. However, unless these involved a comparison against an* in vitro* reference method they were not considered further here. For the same reason we excluded several articles that compared different formulae used to calculate the PGV from standard imaging measurements [8–10] and one study that compared* in vivo* and* ex vivo* MRI measurements (all showing high correlation) [11]. We excluded many articles describing other aspects of the measurement of PGV, such as interobserver variation, or the ability to detect diseases.

No source data extraction for meta-analysis was attempted. Assessment of publication bias was not considered to be necessary. However, the tools for reporting reviews and particularly the QUADAS-2 tool encourage review authors to develop review-specific bias and quality assessments [6]. We considered that the authors of each study might report more favourable results if they were performing most of the imaging themselves, or if those undertaking the reference measurement were not blinded to the results of the imaging. Thus, a bias score was derived with a total score 0-2, a higher score indicating greater potential for bias. The quality of each study was also assessed by considering the imaging measurement (using either a planimetric calculation or autosegmentation method), the reference measurement (using a fresh specimen that had the seminal vesicles removed), the number of patients (more than 50), and whether both concordance and correlation were considered (total score 0 to 4, a higher score indicating higher quality).

## 3. Results

**Figure 1:**
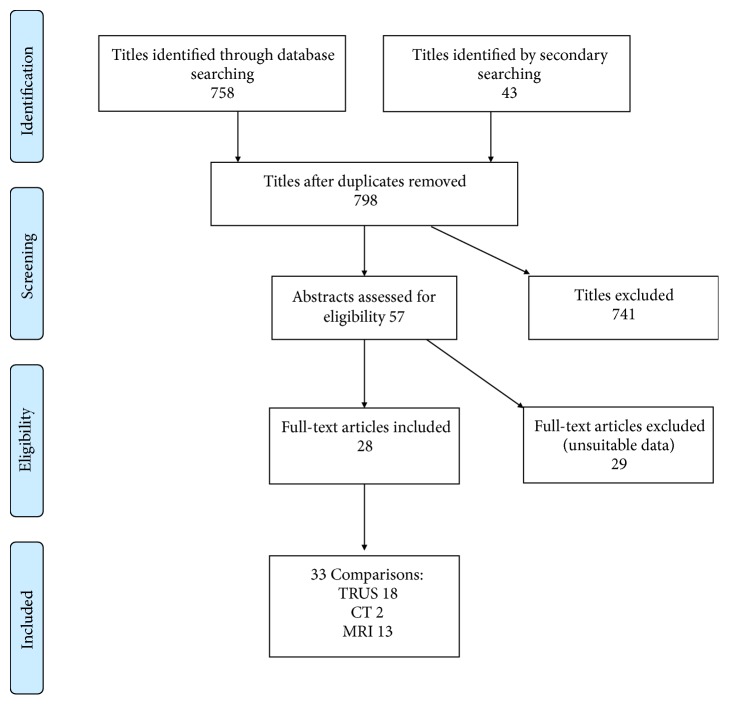
Results of the search strategy.

The search strategy initially generated 758 titles. Selected abstracts were reviewed by both authors blindly, but only 57 were considered relevant. Complete text versions of those articles were obtained, but only 11 had usable data. Secondary searching through 43 titles generated a further 17 articles, identifying a total of 28 articles. Some of these reported imaging measurements from more than one imaging method, describing a total of 33 comparisons between the PGV measured by an imaging method and by the reference method. The search strategy is described in Figure 1.

The 28 articles described studies with a wide variety of sample sizes (5 to 1844 patients) but had a combined total of 7768 patients. The patients were from countries all over the world, mostly USA and Korea but also five different European countries and Australia. The dates of publication were well spread across the range of dates, from 1995 to 2018. The results were tabulated depending on the imaging method used, as shown in Tables 1 (US), 2 (CT), and 3 (MRI). Ages, weights, and volumes were rounded up or down to the nearest whole numbers.

Two articles included both US and CT imaging methods, and these appear in both Tables 1 and 2 [26, 28]. Four articles included both US and MRI imaging methods, in three of these articles both imaging methods were compared with the reference standard, so all three articles appear in both Tables 1 and 3 [20, 22, 29]. In the fourth article, the TRUS measurements were not compared with a reference standard so the results only appear in the table relating to MRI scans, Table 3 [39].

The 18 articles that related to the use of US are shown in Table 1. They were published between 1995 and 2016 and included a total of 4792 patients. All of these used TRUS, but two also used SPUS [26, 28]. The correlation coefficients most commonly fell in the range of 0.70-0.90, indicating high levels of correlation.

Only two articles were related to the use of CT [26, 28]. They involved 223 patients in total and were published in 2013 and 2014. Both of these also included results about TRUS, as shown in Table 2. Only one of these [28] recorded a correlation coefficient at 0.78. Both indicated that the CT volumes were generally larger than TRUS and less accurate. Both also assessed SPUS and found little difference between SPUS and TRUS.

There were 13 articles that related to the use of MRI as shown in Table 3. They included 3388 patients and were published between 2003 and 2018. Correlation coefficients commonly lay between 0.8 and 0.96, a slightly higher range than TRUS and CT. Four articles that described both MRI and TRUS all indicated slightly better results for MRI [13, 20, 22, 29].

While reviewing the articles we made various observations about the methods that were used. The articles often applied geometric terms to describe the shape of the prostate in order to calculate the PGV using each imaging method. The term “ellipsoid” was often used, which is a 3-dimensional volume with three perpendicular axes. The term “spheroid” was sometimes used, meaning that two of the axes are identical. The term “prolate spheroid” was also sometimes used, meaning that these two axes are shorter than the lengthened third axis (rugby ball shape). To convert the measurements of the three axes to a volume, the ellipsoid calculation (EC) was often made by applying the standard formula (height × length × width × *π*/6). A wide variety of modifications to this were used. Other articles often used a planimetric calculation (PC or volumetry), which involves contouring the periphery of the gland on consecutive 3-5 mm slices, either axial or sagittal, and summating the series of volumes.

The reference tests were laboratory (*in vitro*) assessments of prostatectomy specimens which could be analysed by either weighing the specimen or measuring displacement. Weighing was done either by weighing the fresh specimen or after fixation with formalin. In some articles, the specimen was weighed after removal of fat, seminal vesicles or remnants of the vasa deferentia. Some articles subtracted a standard weight for the seminal vesicles from the prostate weight, which might be expected to be more inaccurate in prostates that were unusually large or small. Also in some articles, the weight of the prostate was converted to a volume by applying standard values for the specific gravity of prostate tissue (1.05 g/mL). In some articles, the volumes were identified by displacement of fluid or by measuring the maximum dimensions and using these to calculate an ellipsoid. These variations in the imaging and reference tests were recorded in the tables. These variations in methodology appeared to make little or no difference to the accuracy measures.

The bias and quality scores revealed that no articles were completely free of bias as in nearly all of the articles the authors conducted the imaging assessment themselves and it was rarely stated that those undertaking the reference measurement were blinded to the results of the imaging measurement. Quality scores generally improved with the date of publication. There was no indication that bias or quality played a major role in influencing the reported accuracy of the imaging methods used for PGV measurement.

## 4. Discussion

We found that no previous review of this topic had been performed and that the accuracy of imaging as a method of measuring the PGV was most commonly defined by correlation statistics that were generally moderate to high, most commonly between 0.70 and 0.96. Overall these results suggest that imaging is an accurate test for quantitatively measuring PGV and could be used in a study of the effects of NADT. Of the various imaging methods, TRUS was the most commonly studied. It had been studied long before our cut-off date of 1995, but the accuracy could be expected to depend on technical factors such as the image acquisition time and the resolution of the image, which have improved over time. Immobilisation of the patient may also have improved, especially if the lithotomy position is used rather than the lateral decubitus position. There were only two CT articles, both of which suggested that the scan overestimated the PGV. MRI articles only appeared after 2003, but MRI appeared slightly more accurate, including all three articles that directly compared TRUS and MRI. TRUS could be expected to be more operator dependant than MRI and TRUS measurements are likely to be affected more by pressure on the prostate from the balloon than by an endorectal coil (ERC), although the ERC also involves a balloon that can affect the volume [40]. MRI software may include multifeature active shape models (MFA's) which provide an accurate, automated method of planimetric measurement [32]. The software may also include sophisticated mechanisms for aligning the prostate images* ex vivo* with* in vivo* images, providing an additional means of assessing the PGV [41].

For those articles that described the EC method of volume measurement, there were inconsistent findings about which planes or axes to use. Some showed that the dimensions of the prostate measured on a midsagittal plane were more accurate than an axial plane on TRUS [22] and MRI [30] although an earlier TRUS study had found no difference [16]. Several articles showed that the PC method was more accurate than EC for TRUS and MRI [22, 30, 32, 38]. When PC was done by automated methods, these were just as accurate and could be recorded faster than by manual methods [32, 33, 39].

Regarding the tendency to over or underestimate the PGV, seven articles described this tendency without dividing the patients into those with larger or smaller prostates and found mixed results. For TRUS, four were underestimated while one was overestimated. With CT both were overestimated, while with MRI four were underestimated. There were four articles that divided patients into those either above or below their median values and three found the imaging tended to overestimate smaller glands and tended to underestimate larger glands, while in the remaining one it was the reverse. The underestimation of larger PGVs was the most consistent finding. The optimal way to assess the over and underestimation with volume is with Bland-Altman statistical methods, as these can show how the pattern changes across the range of volumes [42, 43]. There were few articles in this review that used this method [32, 36].

Our review had some limitations. Firstly, the methods used to perform the imaging, to calculate the volume, and to compare it with the reference methods all varied widely, making it difficult to combine them. Secondly, there were variations in the reference test methods used, with many using specimen weight rather than volume. Thirdly, none of the articles were completely free of bias, and none achieved maximum potential quality. However, none of these limitations seem likely to affect the conclusions we have drawn.

Future studies into the measurement of the PGV should use the MRI when the highest level of accuracy is needed using planimetric methods of calculation. Ideally a 3-tesla machine would be used to achieve optimal image quality and without an ERC as that can distort the PGV. The assessment of the volume of individual zones within the prostate could be studied as these can be affected differently by different diseases and treatments. When assessing a method of measurement of the PGV, multiple operators and blinding should be incorporated to avoid bias. The reference method would ideally involve assessment of the PGV by displacement as soon as the prostate is removed, avoiding the effects of shrinkage during fixation and avoiding the need for a volume conversion factor when weight is used. Extraneous tissue should be removed, including the seminal vesicles and remnants of the vasa deferentia. Measures of correlation and concordance should be included, and Bland-Altman plots should be presented to graphically demonstrate agreement, including under and overestimation.

## 5. Conclusions

Our study suggests that the use of imaging to measure the PGV is still a topic of significant interest and that no previous systematic reviews have been undertaken. The correlation of the PGV measured by imaging with the reference methods was in the range of a distribution from 0.70 to 0.96, which is accurate enough for some of the purposes that require quantitative PGV measurements. MRI was slightly more accurate than the other methods.

## Figures and Tables

**Table 1:** Summary of articles measuring the PGV by TRUS in chronological order.

First author, Year of publication, Country	Number of patients, Age	TRUS Imaging details, Mean volume	Reference method, Mean volume	Reference method details	Correlation data	Concordance Data and over/under estimation	Other comments	Scores for Bias (0-2) and Quality (0-4)
Wolff [12] 1995 Germany	25 pts, age NS	EC, Mean NS	Specimen weight, SGF applied, mean 36gm	SV weight subtracted	Linear regression R=0.83 P<0.0001	NS	Two methods of EC compared, NSD	B2 Q0

Tewari [13] 1996 USA	48 pts Age NS	EC mean 60gm	Weighed after fixation, SGF applied, mean 65 gm	SV removed	NS	Students t-test p=0.04 PGV was underestimated by about 10%	Also used MRI but not compared with reference.	B2 Q1

Matthews [14] 1996 USA	100 pts Age NS	EC Mean 36mL	Mean 45 mL EC from measurements	Within 1 hr of excision	NS	Students t-test P<0.01 PGV was underestimated if <30mL and overestimated if >30mL		B2 Q1

Zlotta [15] 1999 Belgium and Austria	36 pts Age NS	EC Mean 29 mL	Weighed Mean 34 mL	Details NS	Pearson's R=0.78 P<0.001	Students t-test p=0.004	TZ volume measurement was more accurate than whole prostate	B2 Q0

Park [16] 2000 South Korea	16 pts Mean 62 yrs	EC, mean 30 mL transaxial and 33 mL midsagittal	EC from specimen, mean 32 mL	Within 1 hr of excision	0.71 Midsagittal 0.83 Transaxial Method NS	Student's t-test NSD	AP measured in two planes, NSD	B2 Q1

Freedland [17] 2005 USA	753 pts Age NS	Details NS	Weight, otherwise details NS	Included SV and vasa tips	Spearman r=0.71 P<0.001	NS	From a larger study of 1602 RP pts in the SEARCH database, mean age 63 yrs, mean specimen weight 44gm	B1 Q1

Loeb [18] 2005 USA	1844 pts Mean 65 yrs	EC Mean 40gm	Mean wt 50 gm	Included SV	Spearman's R=0.65	PGV was generally underestimated, more accurate with smaller PGV	TRUS better than DRE	B1 Q2

Cabello-Benevente [19] 2006 Spain	33 pts Age NS	EC Mean 39cc	Weight 54 gm	Details NS	Pearson r=0.79	Student's t-test P=0.001 Underestimated by 29%		B2 Q1

Lee [20] 2006 Korea	73 pts, age NS	EC Mean 39cc	Fresh weight within 1 hr, SGF applied, mean 37cc	SV removed	Pearson r=0.88 P< 0.001	Overestimated if <35cc, underestimated if >35cc	Also tested MRI, which was more accurate	B2 Q4

Sajadi [21] 2007 USA	497 pts Mean 60yrs	EC Mean 37.4cc	Specimen weight mean 45 gm	Fresh Weight included SV	Spearman's R=0.692, p<0.001	Usually underestimated	VA study	B1 Q3

Jeong [22] 2008 Korea	21 pts, mean 66 yrs	EC, Means 42-51 mL	Fresh specimen within 1 hr, displacement method, mean 40mL	SV removed	Linear regression, R=0.90-94	Students t-test P=0.1-<0.001 Axial and midsagittal measurements of AP were compared, axial better for TRUS	Also used MRI with both EC and PC, where Midsagittal and PC most accurate	B2 Q3

Rodriguez [23] 2008 USA	124 pts Age NS	EC Mean NS	Displacement method and weight (together correlated 0.997).	Defatted but SV attached.	Correlations not given but only 24% within +/- 10%	Underestimated wt in all size categories	No mean values given	B1 Q2

Acer [24] 2010 Turkey	5 pts Mean 60 yrs	EC Mean vol 43 cc	Fluid displacement Mean 53 cc	SV removed	Kruskal Wallis P = 0.677 (NSD)	21% underestimation		B2 Q2

Hong [25] 2012 Australia	236 pts Mean 61 yrs	EC 37 mL	Weight post Formalin fixation 46 mL	SV removed	Spearman r=0.74	Concordance coefficients also provided 0.31-0.46, considered poor	Also performed EC on specimens, median 32 mL, concluded weight more useful	B1 Q3

Varkarakis [26] 2013 Greece	60 pts mean 64 yrs	Both TRUS and SPUS Both EC, means 45-50 cc resp	Displacement of fresh specimen, mean 45 cc	SV and vas removed	NS	SPUS overestimated PGV, TRUS NSD	Also used CT	B2 Q2

Bienz [27] 2014 Canada and USA	440 pts Age NS	EC 4 Volume categories	Weighed before fixation	Details NS	Pearson improved with volume r = 0.17-0.84 P= 0.056-<0.01	ANOVA PGV was underestimated <30 and overestimated >80cc, avg absolute error 39%	Median lobe made no difference	B2 Q3

Kilic [28] 2014 Turkey	163 pts, mean age 64 yrs	EC TRUS and SPUS, means 51 and 50 mL respectively	Fresh weights, Mean 55 gm	SV included SGF applied	ICC 0.84-0.90	Both TRUS and SPUS underestimated the PGV TRUS slightly better than SPUS (NSD)	Also used CT, TRUS and SPUS more accurate	B2 Q2

Paterson [29] 2016 Canada	318 pts Mean 63 yrs	EC Mean 39cc	Fluid displacement method. Mean 37cc	Prostate weight also used (ICC=0.96)	ICC 0.74	Underestimated on average by 3cc	MRI slightly more accurate	B1 Q3

Pts: patients, Yrs: years of age, TRUS: transrectal ultrasound, SPUS: suprapubic ultrasound, EC: ellipsoid calculation, PC: planimetric calculation, NS: not stated, VA: Veterans Affairs, SV: seminal vesicles, TZ: transitional zone, MRI: magnetic resonance imaging, CT: computer tomography, AP: anteroposterior, ICC: intraclass correlation coefficient, SGF: specific gravity factor (1.05 g/mL), and SEARCH: shared equal access regional cancer hospital.

**Table 2:** Summary of articles measuring the PGV by CT.

First author, Year of publication, Country	Number of patients, Age	CT Imaging details Mean volume	Reference method Mean volume	Reference details	Correlation data	Concordance Data and over/under estimation	Other comments	Scores for Bias (0-2) and Quality (0-4)
Varkarakis [26] 2013 Greece	60 pts, Mean 64 yrs	EC, Mean 54 cc	Displacement of fresh specimen, Mean 45 cc	SV and vas removed	NS	Overestimated PGV	Also used TRUS and SPUS, CT larger and less accurate	B2 Q2

Kilic [28] 2014 Turkey	163 pts, Mean age 64 yrs	EC, Mean 63 mL	Fresh weights, Mean 55 gm	SV included SGF applied	ICC 0.78	Overestimated on average by 15%, better agreement for larger PGV	Also used TRUS and SPUS, CT larger than both p<0.001	B2 Q2

Pts: patients, Yrs: years of age, TRUS: transrectal ultrasound, SPUS: suprapubic ultrasound, EC: ellipsoid calculation, PC: planimetric calculation, NS: not stated, SV: seminal vesicles, ICC: intraclass correlation coefficient, and SGF: specific gravity factor (1.05 g/mL).

**Table 3:** Summary of articles measuring the PGV by MRI in chronological order.

First author, Year of publication, Country	Number of patients, Age	MRI Imaging details, Median volume	Reference method, Median volume	Reference details	Correlation data	Concordance Data and over/under estimation	Other comments	Scores for Bias (0-2) and Quality (0-4)
Sosna [30] 2003 USA	11 pts, Mean 59 yrs	EC, PC, and *ex vivo* PC Mean volumes 26-31 EC, 37 PC, 34 mL *ex vivo,*3T MRI, no ERC	Fresh specimen weighed, SGF applied, Mean 40mL	SV removed	Linear regression R=0.32-0.75 for EC, 0.65 for PC in vivo, 0.86 for PC *ex vivo*		6 combinations of various axes used for EC, best was sagittal for AP and SI, axial for RL	B2 Q2

Lee [20] 2006 Korea	73 pts, age NS	EC, Mean 38cc 3T or ERC NS	Fresh weight within 1 hr, SGF applied, Mean 37cc	SV removed	Pearson R=0.96 P< 0.001	Overestimated if < 35cc, underestimated if >35cc	Also tested TRUS, but MRI more accurate	B2 Q4

Jeong [22] 2008 Korea	21 pts, Mean 66 yrs	EC and PC, Means 41-51 mL, ERC used, 3T NS	displacement method, Mean 40mL	Fresh specimen within 1 hr, SV removed	Linear regression, R=0.84-92	Students t-test P=0.03-0.70	PC most accurate	B2 Q3

Kwon [31] 2010 Korea	579, Mean 64 yrs	EC, Mean 32 mL, 1.5T MRI, ERC NS	Fresh weight, Mean NS	SV removed	Pearson R=0.69 P< 0.001	NS		B2 Q2

Bulman [32] 2012 USA	91 pts, Mean 59 yrs	mpMRI EC, PC (manual and MFA). ERC, 3T, Mean 41-45 mL	Freshly weighed, Mean 50 mL	Average weight of SVs subtracted	Wilcoxon signed rank test and linear regression 0.78-0.90	Bland-Altman plots, 92-97% within limits of agreement. All of the MRI methods underestimated the volume by around 15%	Multiple readers used, MFA similar in accuracy to manual planimetry, both more accurate than EC	B2 Q4

Turkbey [33] 2012 USA	98 pts, Median 61 yrs	EC, PC and automated PC, Means 29-48 cc 3T, ERC	Fresh specimen weight, Mean 52 cc	Included SVs	Pearson r=0.86-0.91 P<0.0001	Partial and full Dice similarity coefficient 0.85-0.92	Autosegmentation faster than manual PC	B2 Q3

Karademir [34] 2013, USA	61 pts, Median 64 yrs	Automated volume calculation, Mean 46 cc, 1.5T mpMRI, ERC	Weight from pathology reports, mean 50cc	Standard SV weight subtracted	Pearson r=0.94 P<0.0001	Underestimated by 10% on average		B2 Q2

Hong [35], 2014, USA	1756 pts, Median 59 yrs	EC, Median 31mL 3T NS, ERC NS	Weight from pathology report, Mean NS	Details NS	Pearson R=0.82, p< 0.0001	NS	Higher grade cancer associated with smaller volume	B2 Q1

Le Nobin [36], 2014, USA	37 pts, Mean 60 yrs	PC Mean 47 mL 3T mpMRI, ERC NS	Post fixation, Mean 47 mL	Details NS	NS	Bland Altman 95% limits -7 to +8 mL		B2 Q2

Chernyak [37], 2015, USA	49 pts, Mean 59 yrs	EC, either 1.5T or 3T With and without ERC, Means 46 and 51 cc	Weight retrospectively collected from report, Mean 55 gm	Included SV	ICC improved with ERC 0.90-0.96, mainly due to AP measurement	MRI underestimated weight, more so with ERC (9 vs 4 gm)		B1 Q2

Mazaheri [38], 2015, USA	195 pts, Median 62 yrs	EC and PC, Median 42 cc for both, 3T MRI, ERC	Fresh weight from pathology report, Median 52 cc	Standard SV weight subtracted, applied SGF	Lin CCC used to assess correlation and concordance	Lin CCC = 0.85 (EC) and 0.87 (PC), both underestimated by approx. 10mL		B2 Q3

Paterson [29], 2016, Canada	318 pts Mean 63 yrs	EC Mean 39cc	Fluid displacement method. Mean vol 37cc	Also prostate weight (ICC=0.96)	ICC 0.83	Overestimation more common when a median lobe was present	Also used TRUS, MRI slightly more accurate,	B1 Q3

Bezinque [39], 2018, USA	99 pts, Median 63 yrs	Various EC and PC methods, Medians 35 to 49, 3T mpMRI, No ERC	Specimen wt and volume, Medians 37-54 mL	Details NS	ICC 0.66-0.73	NS	MRI with segmentation was considered the reference	B1 Q2

Pts: patients, Yrs: years of age, TRUS: transrectal ultrasound, EC: ellipsoid calculation, PC: planimetric calculation, NS: not stated, SVs: seminal vesicles, TZ: transitional zone, mpMRI: multiparametric magnetic resonance imaging, MFA: multifeature active shape model, ERC: endorectal coil, 3T: 3-tesla, AP: anteroposterior, ICC: intraclass correlation coefficient, SGF: specific gravity factor (1.05 g/mL), CC: craniocaudal, SP: specific gravity, SEARCH: shared equal access regional cancer hospital, and Lin CCC: Lin's concordance correlation coefficient.
